# Anti-HER2 Drugs for the Treatment of Advanced HER2 Positive Breast Cancer

**DOI:** 10.1007/s11864-023-01137-5

**Published:** 2023-10-25

**Authors:** Malwina Stanowicka-Grada, Elżbieta Senkus

**Affiliations:** Department of Oncology and Radiotherapy, Medical University of Gdansk, 17 Smoluchowskiego Street, Gdansk, 80-214 Poland

**Keywords:** Metastatic breast cancer (MBC), HER2 (human epidermal growth factor receptor 2) overexpression, Monoclonal antibodies, Tyrosine kinase inhibitors, Antibody-drug conjugates

## Abstract

Approximately 15–20% of breast cancers (BC) demonstrate HER2 overexpression/gene amplification. Historically, before the era of HER2-directed therapies, this subtype was associated with poor prognosis. Anti-HER2 agents dramatically changed the natural course of disease and significantly prolonged patients’ survival. In recent years, a number of new anti-HER2 therapies have been developed, and their approvals offer new therapeutic options for patients with advanced HER2-positive breast cancer. At present, HER2 pathway blocking drugs used in the treatment of metastatic breast cancer worldwide include trastuzumab and pertuzumab in the first-line treatment; trastuzumab deruxtecan and trastuzumab emtansine in the second line; and tucatinib, neratinib, lapatinib, and margetuximab in further lines of treatment of advanced HER2 positive breast cancer. Additionally, there are many clinical trials underway evaluating drugs blocking the HER2 pathway in advanced disease setting. This article presents new treatment options, discussing the most important findings from clinical trials and real-world reports, clinical benefits and risks of treatment, as well as efficacy of re-treatment with trastuzumab in metastatic breast cancer. New data challenge the current standards, and a number of questions arise regarding the optimal sequence of anti-HER2 targeted therapies, the optimal combination, including endocrine agents in luminal HER2 positive tumors and treatment of special patient population such as patients with brain metastases (BM).

## Introduction

Breast cancer is the most common cancer in women and the number one cause of cancer death among women globally [1].

Despite the continuous progress and increasing availability of adjuvant therapies and thus better outcomes in early breast cancer, still 20–30% of patients treated with curative intent will develop distant metastases over the disease course. Additionally, approximately 5–10% of patients present with metastatic disease at the time of the diagnosis [2].

Treatment selection in advanced breast cancer is based on a patient’s and disease characteristics, including, among others, tumor burden, previous treatments, and tumor phenotype, in particular estrogen and progesterone receptor expression and HER2 status [3]. Approximately 15–20% of breast cancers demonstrate HER2 receptor overexpression/gene amplification [4]. Overexpression of HER2 protein or amplification of the gene encoding it is both predictive and prognostic. Historically, until the introduction of drugs blocking the HER2 pathway, the prognosis of patients with HER2-positive advanced breast cancer was dismal. The introduction of anti-HER2 therapies significantly improved the outcomes in this patient subgroup, making them comparable or even superior to those seen in HER2-negative disease [5].

## Monoclonal antibodies

### Trastuzumab

Trastuzumab is a humanized monoclonal antibody that binds selectively to HER2 receptor, inhibiting the proliferation of cells that overexpress HER2. It is also a mediator of antibody-dependent cellular cytotoxicity (ADCC). The pivotal H0648g phase 3 study, which compared the efficacy of chemotherapy alone to combination of trastuzumab and chemotherapy, included 469 patients with HER2-positive metastatic breast cancer, of which 234 received chemotherapy and 235—chemotherapy with trastuzumab [6]. Patients exposed to anthracyclines in the adjuvant setting were treated with paclitaxel; those not previously exposed to anthracyclines received them on study. The addition of trastuzumab to chemotherapy significantly increased progression-free survival (PFS) (median 7.4 vs. 4.6 months; *p* < .001), duration of response (median 9.1 vs. 6.1 months; *p* < .001), overall response (50% vs. 32%, *p* < .001) and overall survival (OS) (median 25.1 vs. 20.3 months; *p* = .01). The most important adverse event was cardiac disfunction, in particular among patients treated with combination of anthracycline and trastuzumab. Trastuzumab was approved by EMA (European Medicines Agency) in 2000 for the treatment of HER2-positive MBC, as a monotherapy after at least two line chemotherapy regimens or in combination with paclitaxel/docetaxel in patient who have not received chemotherapy before.

A phase 2 M77001 study [7] compared the efficacy and safety of first-line trastuzumab with docetaxel vs. docetaxel alone in patients with HER2 metastatic breast cancer. Patients enrolled to the trastuzumab plus docetaxel group experienced significant improvements in overall response rate (61% vs. 34%; *p* = .0002), progression free survival (median 11.7 vs. 6.1 months; *p* = .0001), and overall survival (median 31.2 vs. 22.7 months; *p* = .0325). In the chemotherapy alone arm 57% of patients had crossed over to trastuzumab after termination of docetaxel-alone therapy. Although the longest survival was seen in the group with trastuzumab from the beginning of treatment, survival benefits were also seen in the group that crossed-over to trastuzumab (median estimated OS of 30.3 months), while the shortest survival was observed in the docetaxel-alone (no cross-over) group, with median OS of 16.6 months.

The concept of continuing HER2 blockade beyond progression was tested in phase 3 GBG-26 study [8], which included women with HER2 positive metastatic breast cancer, who progressed during trastuzumab-based treatment. Patients were allocated to capecitabine alone or combined with trastuzumab. Unfortunately, the study was closed prematurely due to slow accrual after publication of the results of a lapatinib trial in similar patient population. In spite of that, the study showed significant improvement in time to progression (TTP); median TTP in the capecitabine group was 5.6 months vs. 8.2 months in trastuzumab plus capecitabine group (hazard ratio, HR 0.69; *p* = .0338). No significant overall survival benefit was observed, with median OS of 20.6 and 24.9 months for capecitabine and capecitabine plus trastuzumab, respectively (HR 0.94; *p* = .73) [9]. Additionally, in the subgroup which received 3rd line therapy with anti-HER2 agent, the post-progression survival was 5.5 months longer compared with group without targeted post-progression treatment (median 18.8 months vs. 13.3 months, HR 0.63; *p* = .02), suggesting benefit from further continuation of HER2 blockade.

Some real-world studies suggest that patients may have survival benefits from continuation of trastuzumab beyond progression (TBP). In a German real-world prospective study, 261 patients with advanced/metastatic HER2-positive breast cancer continued TBP (for more than 1 month) after first-line trastuzumab therapy and 157 patients discontinued (discontinuation within 30 days) [10]. Survival benefit was seen in the group continuing trastuzumab, with median OS of 22.1 months, compared to 14.9 months in the group stopping trastuzumab (HR = 0.64; *p* = 0.00021), supporting the strategy of continuing HER2 blockade beyond progression.

As many data suggest that luminal and non-luminal HER2 positive breast cancers are different entities with regard to their biology and response to therapies [11, 12] and hormonal receptors are important targets also in HER2-positive cancers, efficacy of the combination of endocrine therapies (ET) with HER2 directed agents was an obvious and relevant question to ask in clinical trials addressing this population. However, only few of such studies have been reported. The combination of endocrine therapy and trastuzumab was tested in the phase 3 TAnDEM study [13]. Patients were randomly allocated to anastrozole alone or combined with trastuzumab; those in anastrozole alone arm had an option to switch to trastuzumab after progression. Patients treated with trastuzumab plus anastrozole had improved PFS compared with anastrozole alone (median PFS 4.8 vs. 2.4 months, HR 0.63, *p* = .0016); however, OS was not statistically different (median 28.5 months in combination arm vs. 23.9 months in anastrozole alone arm, *p* = .325). The lack of significant OS differences could have been related to the possibility of cross-over for patients in the endocrine therapy alone arm—about 70% of patients in the control group were further treated with trastuzumab at progression. Additionally, the study was not powered for OS analysis. The results of exploratory analyses comparing patients allocated to combination arm with patients treated with anastrozole who did not cross over to trastuzumab after progression demonstrated numerical OS prolongation in the combination arm (median 28.5 months vs. 17.2 months, p=.218). Adverse events were more frequent in the combination treatment, the most common being fatigue, vomiting and diarrhea but no new or unexpected toxicities were identified. The short PFS has been explained by aggressive phenotype of HER2/hormone receptor co-positive tumors compared with HER2 negative and hormone receptor positive cancers.

The efficacy and safety of chemotherapy, endocrine or other anti-HER2 targeted therapy alone or in combination with trastuzumab in first line or after progression was evaluated in a metanalysis [14] of seven trials (Slamon 2001; Marty 2005; Gasparini 2006; Kaufman 2009; von Minckwitz 2009; Blackwell 2010; Huober 2012), with a total of 1497 patients. Trastuzumab was combined with chemotherapy in 4 trials, with endocrine therapy—in 2 trials and with lapatinib—in 1. In 5 studies, trastuzumab was administered in the first-line setting, and in 2 studies was given beyond progression on trastuzumab. Overall, statistically significant PFS improvement was demonstrated among patients treated with trastuzumab containing regimens (HR 0.61, *p* < .00001). PFS prolongation was also demonstrated in an analysis stratified by type of therapy: in taxane-containing (HR 0.53), in anthracycline-containing (HR 0.78), in aromatase-inhibitor containing (HR 0.64) regimens, and with other types of treatment (HR 0.72) and by treatment lines: both with first-line trastuzumab (HR 0.56, *p* < .00001) and beyond progression (HR 0.72, *p* = .001). The results of the analysis evaluating the differences between lines of treatment indicate a greatest benefit of trastuzumab in the first line (*p* = 0.04). All 7 trials demonstrated also improvement of objective response rate (ORR), (risk ratio (RR) 1.58, *p* < .00001), both in first-line setting and beyond progression and OS—in whole analyzed population (HR 0.82, *p* = .004) among patients treated in first-line (HR 0.79, *p* = .006) and with taxane-containing regimens (HR 0.80, *p* = .04). The safety profile suggested an increased risk of congestive heart failure (RR 3.49, *p* = .0009) and reduction of left ventricular ejection fraction (RR 2.65, *p* = .006).

### Pertuzumab

Pertuzumab is a humanized monoclonal antibody that binds to subdomain II of the HER2 receptor and blocks heterodimerization and signal transduction via MAPK (mitogen-activated protein kinase) and PI3K (phosphoinositide 3-kinase)/protein kinase-B (AKT) pathways essential for tumor growth (in contrast to trastuzumab, which binds to subdomain IV and does not inhibit heterodimerization of HER2/HER3 and HER2/HER1). The phase 3 CLEOPATRA trial [15], which enrolled 808 patients with metastatic or inoperable, locally advanced HER2-positive disease, compared the combination of trastuzumab with docetaxel and pertuzumab to trastuzumab plus docetaxel and placebo in the first-line setting. The experimental treatment resulted in prolonged PFS (median 18.7 months vs. 12.4 months, HR 0.68; *p* < .001), and overall survival (median 57.1 months vs. 40.8 months, HR 0.68, *p* < 0.001). Most important pertuzumab-related adverse events included diarrhea, leukopenia, and allergic reactions. No increase in cardiac toxicity, including left ventricular dysfunction was observed. Pertuzumab was approved by EMA in 2013 and has become standard first-line treatment in combination with trastuzumab and docetaxel in HER2-positive MBC patients not previously treated with HER2 pathway blockers or with relapse more than 6 months after completion of perioperative trastuzumab-based treatment.

The safety and efficacy of pertuzumab and trastuzumab combined with investigator selected taxane in first-line treatment of HER2-positive advanced breast cancer were confirmed in the single-arm PERUSE study [16], with the safety profile (which was the primary endpoint) being consistent with earlier study reports (most common grade ≥ 3 adverse event were neutropenia and diarrhea). After 5.7 years follow-up, median PFS was 20.7 months irrespective of hormone receptor status and type of taxane (paclitaxel, docetaxel or nab-paclitaxel), and median OS was 65.3 months, also regardless of taxane used. Longer OS was noted among patients with HR-positive disease and the shortest PFS and OS were observed in the group of trastuzumab-pretreated patients with visceral metastasis.

The PHEREXA phase 3 study [17] was conducted in patients with HER2 positive metastatic breast cancer, who progressed during or after trastuzumab in adjuvant or first-line metastatic setting and received a prior taxane. Two hundred twenty-four patients were allocated to trastuzumab and capecitabine and 228—to dual anti-HER2 therapy with trastuzumab and pertuzumab, combined with capecitabine. Patients in the pertuzumab arm did not experience significant improvements in PFS; median independent review facility assessed PFS (IRF–PFS), which was the primary endpoint was 11.1 months in pertuzumab arm vs. 9.0 months in the control arm (HR 0.82; *p* = .0731); however, combining HER2-targeting agents numerically increased median OS by 8 months (36.1 months in pertuzumab plus trastuzumab arm, compared with 28.1 months in trastuzumab alone arm, HR 0.68; no *p* value cited). The addition of pertuzumab was well tolerated, rates of adverse events were similar in both groups.

Dual HER2 directed therapy has also been studied in combination with endocrine therapy. A total of 258 women with HER2-positive, HR-positive locally advanced or metastatic breast cancer, who had received no prior systemic therapy for locally advanced or metastatic breast cancer, with the exception of endocrine treatment, were enrolled to the PERTAIN phase 2 trial and the benefits were shown in primary [18] and final analysis [19]. Patients were treated with aromatase inhibitor and trastuzumab with or without pertuzumab; additionally, 146 of them received “induction” chemotherapy for 18 to 24 weeks. PFS, which was the primary endpoint, in final analysis was 4.8 months longer in the dual blockade arm (median 20.6 months in pertuzumab group vs. 15.8 months in trastuzumab alone group, HR 0.67; *p* = .006). Pertuzumab treatment effect was potentially enhanced in patients with no induction—in the group of patients who received “induction” therapy PFS medians were the same in both arms (16.9 vs. 16.9 months, unstratified HR = 0.71, *p* = 0.076), whereas in the group of patients without previous chemotherapy median PFS was 26.6 months and 12.5 months, respectively, in the group with pertuzumab and without it (unstratified HR = 0.68, *p* = 0.067). Prolongation of PFS was also seen in the group of patients with estrogen receptor expression in ≥ 10% of tumor cells—in this group median PFS was 22.5 months, compared with 16.4 months in the group with lower hormone receptor expression (HR = 0.66; *p* = 0.012). OS was similar between arms. A total of 56.7% of the pertuzumab-treated population had grade ≥ 3 adverse events (the most common: hypertension, diarrhea, neutropenia), vs. 41.1% in the trastuzumab alone group.

### Margetuximab

Margetuximab is a chimeric monoclonal antibody targeting HER2 receptor similarly to trastuzumab but with the Fc region designed to increase affinity to the activating subtype of the Fc receptor (FcR), CD16A and decrease affinity for the inhibitory FcR, CD32B [20]. Phase 3 SOPHIA study [21] randomized 536 patients after 2 or more prior HER2-directed therapies for metastatic breast cancer to margetuximab plus chemotherapy vs. trastuzumab with chemotherapy. Median PFS in the margetuximab arm was 5.8 months vs. 4.9 months in the trastuzumab arm (HR 0.76; *p* = .033); in patients with CD16A genotype containing a 158F allele the difference in treatment outcomes was more pronounced—median PFS value was 1.8 months longer in the margetuximab arm (HR 0.68; *p* = .005), suggesting that the presence of a CD16A-158 allele could be associated with benefits of margetuximab over trastuzumab treatment [22]. Confirmed ORR was 22% in the margetuximab arm and 16% in the control arm. The OS results [22] were not statistically different, median OS in the margetuximab group was 21.6 months vs. 21.9 months in the trastuzumab group (HR 0.95; *p* = .620). Genotyping, including an analysis of CD16A (genotypes: FF, F, VV), was possible in 94% of patients; no survival benefits from margetuximab were seen in the CD32A and CD32B groups, while in patients with CD16A-158FF expression preplanned exploratory analysis showed better median OS in the margetuximab group [23.9 months vs. 19.2 in trastuzumab arm (HR 0.72)], and genotype CD16A-15VV was associated with better outcomes in the trastuzumab arm. Safety profiles were similar in both groups. The most common adverse effects during margetuximab treatment included fatigue, nausea, diarrhea, and neutropenia. Margetuximab combined with chemotherapy is approved for treatment HER2 positive MBC in the USA.

## Tyrosine kinase inhibitors

### Lapatinib

Lapatinib is an inhibitor of HER1 and HER2 tyrosine kinases. In the registration phase 3 EGF100151 study lapatinib combined with capecitabine was compared to capecitabine alone in patients with HER2-positive, locally advanced or metastatic breast cancer, pretreated with anthracycline, taxane, and trastuzumab [23]. The median time to progression, which was the primary endpoint, was 8.4 months in combination therapy and 4.4 months in monotherapy arm (HR 0.47; *p* < .001). The most common adverse effects in both groups were diarrhea, hand–foot syndrome, nausea, and vomiting; diarrhea, dyspepsia, and rash occurred more frequently in the combination arm. No symptomatic cardiac events were observed.

Another phase 3 study (EGF104900) compared lapatinib administered alone or in combination with trastuzumab in patients with HER2 positive, trastuzumab-refractory metastatic breast cancer [24]. The results showed that dual blockade is more effective than single agent in terms of PFS (HR 0.74; *p* = .011). Additionally, the addition of trastuzumab to lapatinib significantly prolonged OS (median 14 months in the combination arm vs. 9.5 months in lapatinib alone arm (HR 0.74; *p* = .026). The frequency of adverse events was similar in both arms, apart from diarrhea, which was higher in the combination arm.

The efficacy of adding lapatinib to letrozole was evaluated in a phase 3 (EGF30008) study [25] among patients with metastatic breast cancer positive for hormone receptor and HER2. Two hundred ninety-one postmenopausal women were enrolled to the first-line treatment with lapatinib and letrozole or letrozole and placebo. Reduction in the risk of disease progression was seen in the combination arm, with median PFS of 8.2 months in the lapatinib with letrozole and 3.0 months in the letrozole alone arm (HR 0.71, *p* = .019). The addition of lapatinib also allowed for improvement in the overall response rate from 15 to 28% (odds ratio [OR] 2.2; *p* = .021) and clinical benefit rate from 29 to 48%, respectively (OR 2.2; *p* = .003). No new safety signals were reported.

Further benefits of anti-HER2 treatment in combination with ET were demonstrated in the phase 3 ALTERNATIVE trial [26], where the 355 postmenopausal, HER2- and HR- co-positive MBC patients, pretreated with ET and trastuzumab plus chemotherapy (in neo-/adjuvant/ first-line setting), were randomized to the one of the three arms: dual anti-HER2 blockade (trastuzumab and lapatinib, T + L) in combination with aromatase inhibitor (AI), trastuzumab (T) plus AI and lapatinib (L) plus AI. The primary endpoint has been reached: superior PFS was observed in the dual HER2 blockade group (T + L + AI group vs. T + AI: median PFS 11 vs. 5.6 months, HR 0.62, *p* = 0.0063). No difference in PFS was found in L + AI vs. T + AI group (median PFS 8.3 vs. 5.6 months, HR 0.85, *p* = 0.3159). OS data are immature, but a trend in favor of dual-blockade was observed. The most common (any grade) adverse events were diarrhea (69%, 9%, and 51%, respectively, in T + L + AI, T + AI, and L + AI groups), rash, and nausea, mostly of grade 1 and 2. The frequency of serious adverse events was similar in all three subgroups.

### Tucatinib

Tucatinib is a selective inhibitor of HER2 tyrosine kinase with minimal inhibition of HER1. A phase 1 study showed antitumor activity of tucatinib in patients with HER2 positive, metastatic breast cancer, including brain metastases [27]. In the registration phase 2 HER2CLIMB study [28••], 612 patients with HER2 positive MBC pretreated with trastuzumab, pertuzumab, and T-DM1 were assigned 2:1 to tucatinib or placebo in combination with trastuzumab and capecitabine. Importantly, the patients were allowed to have untreated or previously treated progressing BM. The estimated PFS at 1 year was 33.1% in tucatinib group vs. 12.3% in placebo group and median PFS was 7.8 months vs. 5.6 months, respectively (HR 0.54; *p* < .001). At 2 years, the risk of death in patients from tucatinib arm was reduced by 34%, compared to patients on trastuzumab and capecitabine alone (median OS 21.9 months vs. 17.4 months, respectively, HR 0.66, *p* = .005). Outcomes were also better in the subgroup of patients with BM: in the tucatinib arm median PFS was 7.6 months vs. 5.4 months in the control arm (HR 0.48; *p* < .001). Among all patients with BM at baseline (198 in the tucatinib arm and 93 in the control arm) the risk of progression in the CNS or death in the tucatinib arm was reduced by 68% (median CNS-PFS 9.9 months vs. 4.2 months, respectively, HR 0.32, *p* < .0001) and median OS was 18.1 vs. 12.0 months in the tucatinib and control arms, respectively (HR 0.58, *p* = .005) [29]. In the subgroup with active BM median, CNS-PFS was 9.5 months vs. 4.1 months, respectively (HR 0.36, *p* < .00001) and the risk of death was reduced by 51% in the tucatinib arm vs. control arm (median 20.7 months vs. 11.6 months, HR 0.49, *p* = .004). The main adverse effects of tucatinib included diarrhea, nausea, vomiting, and elevated aminotransferase levels.

The efficacy and safety of tucatinib were also evaluated in phase 3 HER2CLIMB-02 trial, where patients with HER2-positive MBC pretreated with taxane and trastuzumab were allocated to T-DM1 alone or combined with tucatinib. A recent press release (of August 16, 2023) announced that the HER2CLIMB-02 trial met its primary endpoint of PFS improvement; data about secondary endpoint of OS are not yet mature [30].

Tucatinib is approved since 2020 in combination with trastuzumab and capecitabine for patients with HER2-positive locally advanced or metastatic breast cancer pretreated with minimum one anti-HER2-based regiments in the metastatic settings, including patients with brain metastases (FDA registration)/after at least 2 prior anti-HER2 treatment regimens (EMA registration).

### Neratinib

Neratinib is an irreversible tyrosine kinase inhibitor of HER1, HER2, and HER4. The phase 3 NALA trial [31] included 621 patients after at least 2 prior anti-HER2 regimens in metastatic breast cancer, randomly assigned to neratinib plus capecitabine (N + C) or lapatinib plus capecitabine (L + C). The primary endpoints were centrally assessed PFS and OS; secondary endpoints included time to CNS disease intervention, investigator-assessed PFS, objective response rate, duration of response, clinical benefit, safety, and health-related quality of life. Neratinib significantly improved PFS (median 5.6 months in neratinib vs. 5.5 months in lapatinib arm, HR 0.76; *p* = .0059), whereas median OS was 21 months in neratinib group and 18.7 months in the control group, not statistically different (HR 0.88; *p* = .2098). No difference in ORR was observed (32.8% in N + C group vs. 26.7% in L + C group, *p* = .1201); however, in patients enrolled to neratinib arm there were fewer interventions for CNS disease and the duration of response was prolonged from 5.5 to 8.6 months (HR 0.50; *p* = .0004). The most common adverse effect of neratinib was diarrhea. The quality of life was similar in both groups. Based on NALA trial neratinib is approved in the USA for patients who have received two or more lines of therapy in the metastatic setting.

### Pyrotinib

Pyrotinib is an irreversible dual pan-HER receptor tyrosine kinase inhibitor. Promising antitumor efficacy with acceptable tolerance was shown in phase 1 studies [32, 33]. The phase 2 (NCT02422199) Chinese study [34], among patients with HER2 positive MBC, previously treated with anthracyclines, taxanes, and/or trastuzumab, who were randomized to receive pyrotinib or lapatinib both in combination with capecitabine, demonstrated prolongation of PFS, median 18.1 months in pyrotinib group vs. 7.0 months in lapatinib group (adjusted hazard ratio, 0.36; *p* = 0.001), the most common grade 3 to 4 adverse effect was hand-food syndrome in both arms (24.6% vs. 20.6%, respectively, in pyrotinib and lapatinib arm). It should be noted that about half of the patients had not received trastuzumab before in any settings, because of relatively low access to this drug. The phase 3 PHOEBE study [35] in the group of Chinese patients with HER2 positive MBC after progression on trastuzumab and taxanes, also confirmed the effectiveness of the pyrotinib vs. lapatinib, both with capecitabine: PFS was significantly longer in the pyrotinib arm (median 12.5 months vs. 6.8 months, respectively). The most common adverse effects were diarrhea, vomiting, anemia, and neutrophil count decrease. Pyrotinib is approved in second-line setting in China.

## Anti-body drug conjugates (ADC)

### Trastuzumab emtansine (T-DM1)

Trastuzumab emtansine (T-DM1) is an antibody-drug conjugate consisting of trastuzumab covalently linked to the cytotoxic agent DM1 (derivative of maytansine). ADC by internalization and degradation in lysosomes delivers the cytotoxic drug exactly to the cancer cells, where DM-1 is blinding tubulin, which causes cell cycle arrest at G2-M phase and resulting cell death [36]. EMILIA was a phase 3 trial [37] which first demonstrated the efficacy and safety of T-DM1. Nine hundred ninety-one patients with locally advanced or metastatic breast cancer, who progressed during or after taxane and trastuzumab in adjuvant or metastatic setting, were randomly assigned to receive either T-DM1 or standard at that time treatment with lapatinib plus capecitabine. The median PFS was 9.6 months with T-DM1 group vs. 6.4 months in the control group (HR 0.65; *p* < .001), and median OS—30.9 vs. 25.1 months, respectively (HR 0.68; *p* < .001). Grade 3 and 4 adverse events were significantly more common with lapatinib plus capecitabine than in T-DM1 group (57% vs. 41%, respectively). The most common adverse effects in T-DM1 arm included thrombocytopenia (28%) and elevated serum aminotransferase level (AST 22%, ALT 17%), but dose modifications allowed the majority of patients to continue treatment. Left ventricular ejection fraction of 45% or more was maintained in most patients in both arms. The majority of deaths during the study were caused by disease progression and of the 5 deaths due to adverse events, 4 occurred in the lapatinib plus capecitabine group.

In phase 3 TH3RESA study [38], T-DM1 was compared to physician’s choice treatment (TPC) in 602 patients with HER2-positive locally advanced or metastatic breast cancer after at least 2 prior anti-HER2 regimens, including trastuzumab and lapatinib, and previous taxane therapy. The study showed doubling of median PFS from 3.3 months in TPC patients to 6.6 months in patients allocated to T-DM1 (HR 0.528; *p* < .0001). It should be noted that 83% of patients allocated to TPC received one or more HER2-directed agents as the on-study treatment and after progression 47% of TPC patients crossed over to T-DM1. In spite of that patients originally allocated to T-DM1 achieved longer OS compared with TPC patients (median OS 22.7 vs. 15.8 months, respectively; HR 0.68; *p* = .0007). Grade 3 or higher adverse events were more common in TPC group (47% vs. 40%, respectively), serious adverse events were reported in 25% of patients in T-DM1 group vs. 22% patients of TPC group, death due to adverse events—in 2% of patients in both groups.

Safety and efficacy of T-DM1 in first-line setting were assessed in the three-arm phase 3 MARIANNE study [39]. Patients with HER2 positive advanced breast cancer naive to chemotherapy in the metastatic setting were treated with T-DM1 alone, T-DM1 with pertuzumab or taxane plus trastuzumab. Results of T-DM1 and T-DM1 plus pertuzumab arms demonstrated noninferior PFS compared with control groups (median PFS 15.2 months with T-DM1 plus pertuzumab, 14.1 months with T-DM1, and 13.7 months with trastuzumab plus taxane, stratified HR for T-DM1 vs. trastuzumab plus taxane, 0.91; *p* = .31, stratified HR for T-DM1 plus pertuzumab vs. trastuzumab plus taxane 0.87; *p* = .14), but the superiority of T-DM1-based treatment could not be confirmed. PFS was also not improved by the addition of pertuzumab to T-DM1 (stratified HR for T-DM1 plus pertuzumab vs. T-DM1: 0.91). The longest median response duration was seen in T-DM1 with pertuzumab arm (21.2 months), compared with T-DM1 alone (20.7 months) and taxane with trastuzumab (12.5 months) [40]. The incidence of grade 3 or higher adverse events was higher in taxane plus trastuzumab arm (54.1%), compared to T-DM1 (45.4%) or T-DM1 plus pertuzumab arm (46.2%).

Activity of T-DM1 has also been demonstrated in patients with BM, pretreated with HER2-targeted therapy and chemotherapy of advanced breast cancer. In the single-arm phase 3b KAMILLA study [41], among patients with measurable BM the overall response was seen in 21.4% and the clinical benefits rate (defined as complete, partial response or stable disease lasting minimum 6 months)—in 42.9% of patients. Median PFS among patient with baseline BM was 5.5 months and median OS—18.9 months. The adverse events were overall similar in patients with or without BM but nervous system-related adverse events were more common in the BM subgroup.

### Trastuzumab deruxtecan (T-DXd)

Trastuzumab deruxtecan (T-DXd) is an antibody-drug conjugate composed of trastuzumab, a cleavable linker and a cytotoxic topoisomerase I inhibitor with antineoplastic properties, with high drug-antibody ratio (of about 8). First data on T-DXd come from the DESTINY-Breast01 single arm, phase 2 trial, which enrolled 184 female, heavily pretreated patients (median 6 previous treatment lines) with HER2-positive, unresectable or metastatic breast cancer [42]. The updated data presented at the 2020 San Antonio Breast Cancer Symposium [43] after a 20.5 months of follow-up showed objective response rate of 61.4% (95% CI 54.0–68.5%), median progression-free survival of 19.4 months (95% CI 14.1 month—not estimable), median duration of response of 20.8 months and estimated 12- and 18-month overall survival rates of 85% (95% CI 79–90%) and 74% (95% CI 67%-80%) respectively. Grade 3 or higher adverse events occurred in 57% patients; the most common were decreased neutrophil count (in 20.7% of the patients), anemia (in 8.7%), and nausea (in 7.6%). A total of 13.6% of patients developed interstitial lung disease (ILD) and the pulmonary toxicity led to death in 4 patients out of 25 cases of ILD, so close monitoring for pulmonary symptoms is recommended during this treatment. Trastuzumab deruxtecan was approved by FDA in 2019 and by EMA in 2021 for treatment of patients with HER2-positive unresectable or metastatic BC, who have received two or more prior anti-HER2-based regimens in the metastatic settings; following positive results of the DESTINY-Breast03 study FDA and EMA have extended the recommendation also to second-line setting.

The safety and efficacy of T-DXd vs. T-DM1 in second-line setting in patients with HER2 positive metastatic breast cancer after failure of trastuzumab and taxane treatment (about 60% of patients were also pretreated with pertuzumab) were assessed in the DESTINY-Breast03 phase 3 study [44•]. Median PFS, which was the primary endpoint, in the T-DXd arm was 28.8 months, and for T-DM1—6.8 months (HR = 0.33, *p* < 0.0001). ORR was confirmed in 79% of the patients in T-DXd group vs. 35% in control group. Although median OS was not reached in both groups now, the risk of death during T-DXd treatment was reduced by 36% (HR 0.64, *p* = 0.0037) and the benefits in OS were seen across subgroups irrespective of presence of baseline brain metastases, baseline visceral disease, hormone receptor status, and previous treatment with pertuzumab. In patients who initially had brain metastases (16.8% in T-DXd group and 14.5% in T-DM1 group) [45], treatment with T-DXd decreased the risk of progression or death by 75%; the 12-month PFS rate was 72.0% with T-DXd regiment and 20.9% with T-DM1 (HR = 0.27). ORR was improved from 20.5% in T-DM1 arm to 67.4% in T-DXd arm. Intracranial response was also better during T-DXd treatment, with complete intracranial response in 27.8% of patients, compared with 2.8% patients treated with T-DM1. The number of grade 3 or worse drug related adverse event was similar in both groups [44••]. In T-DXd arm, the most frequent treatment-emergent adverse events were gastrointestinal (nausea and vomiting), hematological (neutrophil count decreased and anemia), alopecia, and pulmonary problems. Incidence of interstitial lung disease or pneumonitis in patients treated with trastuzumab emtansine was 3%, and in trastuzumab deruxtecan—15%, but no grade 4 or 5 were encountered in either group.

The efficacy of the T-DXd treatment was also confirmed in the phase 3 DESTINY-Breast02 [46•] study. In the study, 608 patients with unresectable or metastatic breast cancer were enrolled, after progression during or after T-DM1 treatment, in the control group patients received physician’s choice therapy (a combination of capecitabine with either trastuzumab or lapatinib). An objective response was seen among 70% of patients in the T-DXd group and 29% of patients in control group, median PFS was 17.8 vs. 6.9 months (HR 0.36; *p* < 0.0001), respectively, and median OS in T-DXd arm was 39.2 vs. 26.5 months in the TPC arm (HR 0.66; *p* = 0.0021). Adverse events in patients treated with T-DXd were consistent with previous studies; interstitial lung disease was reported in the 10.4% of patients, most of them were grade 1 or 2, but there were two cases of grade 5 toxicity.

The activity of trastuzumab deruxtecan in HER2-positive or HER2-low MBC with stable, untreated, or progressing BMs and/or leptomeningeal carcinomatosis is evaluated in ongoing phase 2, 5-cohort DEBRRAH study (NCT04420598) [47]. Preliminary results were presented for patients with HER2-positive, advanced BC, with non-progressing BMs after local therapy (cohort 1), asymptomatic untreated BMs (cohort 2), and BMs progressing after local therapy (cohort 3). In cohort 1, 16-week PFS, which was primary endpoint, reached 87.5% (*p* < 0.001). In cohorts 2 and 3, the primary endpoint was the intracranial objective response rate (ORR-IC); it reached 50% in cohort 2 and 44.4% (*p* < 0.001) in cohort 3. ORR in patients with measurable intracranial or extracranial lesions at baseline was 66.7% and most of the patients had reduction in tumor size. Toxicity of T-DXd was consistent with prior reports.

Another study demonstrating intracranial effectiveness of T-DXd is the phase 2 TUXEDO-1 trial (NCT04752059) [48], which enrolled 15 patients with HER2-positive MBC, pretreated with trastuzumab and pertuzumab, with newly diagnosed BMs, untreated or progressing after local therapy. The intracranial response rate reached 73.3%. No new safety signals were observed.

The efficacy and safety of T-DXd in HER2 positive MBC is also assessed in the first-line setting (one line of ET for MBC is allowed), with or without pertuzumab, in phase 3 Destiny-Breast09 study (NCT04784715) [49]. The study consists of 3 groups, treated with T-DXd plus placebo, T-DXd plus pertuzumab, or standard of care trastuzumab plus pertuzumab and taxane. Patients are stratified by prior treatment, hormone receptor, and *PIK3CA* mutation status. PFS by blinded independent central review is the primary endpoint; secondary endpoints include PFS by investigator assessment, OS, DOR, ORR, PFS2, health-related quality of life, safety, pharmacokinetics, and immunogenicity. Another ongoing phase 3b/4 study is Destiny-Breast12 (NCT04739761) [50], assessing effectiveness of T-DXd among patients without brain metastases (cohort 1) and with BMs (cohort 2), with pretreated (1-2 lines, excluded previous tucatinib) advanced/metastatic HER2-positive BC. ORR is the primary endpoint in cohort 1, and PFS in cohort 2. Secondary endpoints include OS, DOR, TTP, duration of subsequent therapy, PFS2, safety and changes in symptoms, functioning, and quality of life.

## Future perspectives

Many clinical trials are underway to evaluate new therapeutic options. Early phase studies suggest promising activity of novel ADCs, like RC-48 [51], ARX788 [52], and bispecific antibodies targeting domains II and IV of the HER2 receptor, e.g., ZW25 [53]. The immunogenicity of HER2-expressing tumors offers further promise in the use of immunological checkpoint inhibitors but the results are still unsatisfactory; in the phase 2 KATE2 study, addition of atezolizumab to T-DM1 did not improve patients outcomes [54].

Further perspectives include, among others, use of CDK4/6 inhibitors (in luminal HER2-positive tumors), PI3K inhibitors (in tumors harboring a *PIK3CA* mutation), and anticancer vaccines. The value of the first concept was supported by the findings of the phase 2 MonarcHER study (NCT02675231), which demonstrated numerical prolongation of PFS and OS with the addition of abemaciclib to trastuzumab combined with endocrine therapy [55] and is being tested in the currently ongoing PATINA trial (AFT-38/NCT02947685) testing the addition of palbociclib to combination of ET and anti-HER2 therapy [56].

## Conclusions

Treatment options for HER2-positive metastatic breast cancer have improved significantly in the last 20 years thanks to development of multiple compounds including new antibodies, tyrosine kinase inhibitors, and antibody drug conjugates. Unfortunately, despite increasingly active drugs targeting HER2 pathway, HER2 positive breast cancer remains incurable, although with new therapies, it may convert to a chronic disease at least in some patient subpopulations.

Following the publication of the CLEOPATRA study, a standard-of-care in first-line treatment of HER2-positive metastatic breast cancer is the combination of trastuzumab with pertuzumab and taxane. Until very recently the standard in second line therapy was trastuzumab emtansine, following the publication of the EMILIA study; however, with the recent data from DESTINY-Breast03 trial, T-DXd became the recommended second-line treatment and T-DM1 was moved into further therapy lines. T-DXd should also be considered in the case of BMs, following the publication of DEBRRAH or TUXEDO-1 studies and the subgroup analysis from the DESTINY-Breast03.

There is no standard-of-care in third-line treatment, although a large proportion of patients remain well enough to continue treatment after failure in first- or second-line and a number of valid treatment options exist for these patients, including those with BM, a population typically excluded from clinical studies. Following second line T-DXd several options are available, such as tucatinib (also an option in second line, in particular in patients with untreated/uncontrolled BMs), T-DM1, trastuzumab plus chemotherapy, lapatinib, or margetuximab. In case of specific clinical features such as brain metastases, tucatinib seems to be the preferred choice, albeit no evidence exists on its efficacy in the post-T-DXd setting. Similarly, in case of T-DM1, very little data is available on its use following T-DXd.

Another treatment option for metastatic breast cancer with expression of HR is ET. The co-expression of endocrine receptors and HER2, albeit associated with resistance to hormonal and anti-HER2 therapy, resulting in worse prognosis, creates an opportunity of combination treatment, consisting of anti-HER2 drugs and ET, in particular when dual HER2-blockade is used. In combination with ET, it provides superior efficacy and comprises a good treatment option for patients who are not candidates for chemotherapy.

Development of new anti-HER2 drugs and wide adaptation of the policy of continuing HER2 blockade beyond progression allowed for improvement of outcomes among advanced HER2 positive breast cancer patients, including prolongation of overall survival (allowing for achievement of median OS extending over 60 months [PERUSE]) and improvements in quality of life, at the expense of acceptable treatment toxicity. Importantly, some of these compounds demonstrate activity also for BM, a sanctuary considered resistant to majority of systemic anticancer treatments.
